# Metabolic Effects of Antidepressants; Is It Time to Change the Conversation?

**DOI:** 10.1192/bjo.2022.231

**Published:** 2022-06-20

**Authors:** Anjali Patel, Yathorshan Shanthakumaran, Reshma Rasheed, Imaduldin Nazir

**Affiliations:** 1 New Vision University, Tbilisi, Georgia; 2 Rigg Milner Medical Centre, East Tilbury, United Kingdom

## Abstract

**Aims:**

The incidence of depression has risen both nationally and internationally. The mainstay of management remains referral to IAPT and treatment with SSRI and SNRIs and the rates of prescribing are rising exponentially. During the COVID-19 pandemic, more people faced mental health challenges. In the last ten years, the incidence of SSRI prescribing rose from 6.8% to 100%. A known side effect of antidepressant medication is weight gain, dyslipidemia, increasing risk of impaired fasting glycaemia and diabetes. Our study was conducted to assess the actual risk incurred in our population from the point of starting therapy till date.

**Methods:**

Patients were identified from the GP clinical system (SystmOne) to identify those with a current prescription of antidepressants and antipsychotics. A retrospective analysis of 591 patients' case records was undertaken. Body weight, BMI, fasting glucose, HbA1c, fasting lipids and Q risk were analysed at the time of prescription initiation, post treatment and any rise in cardiovascular risk over a period of years. The data were analysed to see the trajectory of deterioration in metabolic risk. All patients were assessed to ensure they had been signposted and referred to weight management services.

**Results:**

The data show a positive correlation between the onset of antidepressant and antipsychotic prescribing, worsening of BMI, increase of cardiovascular and metabolic risk. The data show an exponential rise in BMI and metabolic risk (cardiovascular Q risk, dyslipidemia, imparied fasting glycaemia, diabetes and ischaemic heart disease) for patients taking SSRI and SNRI within 12 months. This effect continues for the length of the prescribing interval. We also found that with the rise of BMI dose, escalation was common due to reduced effectiveness. The average rise in cardiovascular Q risk average was 14.05% over three years. Patients need careful counselling at the outset and need regular reassessment of metabolic risks at each medication review. Informed consent must be obtained - risks of SSRI, SNRI and antipsychotic risk should be stated.

**Conclusion:**

A known iatrogenic risk of antidepressant medication is weight gain, dyslipidemia, increasing risk of impaired fasting glycaemia and diabetes. Careful counselling and metabolic risk assessment is required when initiating these medications. Throughout the length of prescribing patients need re-assessment of their cardiovascular and diabetes risk with timely referral to weight management services to counterbalance metabolic risks.

